# COVID-19 in patients presenting with malaria-like symptoms at a primary healthcare facility in Accra, Ghana

**DOI:** 10.1371/journal.pone.0298088

**Published:** 2024-02-09

**Authors:** Issabella Asamoah, Mildred Adusei-Poku, Priscilla Vandyck-Sey, Allen Steele-Dadzie, Atta Senior Kuffour, Albert Turkson, Ivy Asantewaa Asante, Kantanka Addo-Osafo, Quaneeta Mohktar, Bright Adu, Yaw A. Afrane, Kwamena W. C. Sagoe

**Affiliations:** 1 Department of Medical Microbiology, University of Ghana Medical School, University of Ghana, Korle-Bu, Accra, Ghana; 2 Korle Bu Polyclinic Family Medicine Department, Korle Bu Teaching Hospital, Accra, Ghana; 3 Department of Virology, Noguchi Memorial Institute for Medical Research, University of Ghana, Legon, Accra, Ghana; 4 Department of Immunology, Noguchi Memorial Institute for Medical Research, University of Ghana, Legon, Accra, Ghana; Food and Drug Administration, UNITED STATES

## Abstract

**Background:**

Malaria is a common and severe public health problem in Ghana and largely responsible for febrile symptoms presented at health facilities in the country. Other infectious diseases, including COVID-19, may mimic malaria due to their shared non-specific symptoms such as fever and headache thus leading to misdiagnosis. This study therefore investigated COVID-19 among patients presenting with malaria-like symptoms at Korle-Bu Polyclinic, Accra, Ghana.

**Methods:**

This study enrolled 300 patients presenting with malaria-like symptoms aged ≥18yrs. After consent was obtained from study patients, two to three millilitres of whole blood, nasopharyngeal and oropharyngeal swab samples, were collected for screening of *Plasmodium falciparum* using malaria rapid diagnostic test, microscopy and nested PCR, and SARS-CoV-2 using SARS-CoV-2 antigen test and Real-time PCR, respectively. The plasma and whole blood were also used for COVID-19 antibody testing and full blood counts using hematological analyser. SARS-CoV-2 whole genome sequencing was performed using MinIon sequencing.

**Results:**

The prevalence of malaria by microscopy, RDT and nested PCR were 2.3%, 2.3% and 2.7% respectively. The detection of SARS-CoV-2 by COVID-19 Rapid Antigen Test and Real-time PCR were 8.7% and 20% respectively. The Delta variant was reported in 23 of 25 SARS-CoV-2 positives with CT values below 30. Headache was the most common symptom presented by study participants (95%). Comorbidities reported were hypertension, asthma and diabetes. One hundred and thirteen (37.8%) of the study participants had prior exposure to SARS CoV-2 and (34/51) 66.7% of Astrazeneca vaccinated patients had no IgG antibody.

**Conclusion:**

It may be difficult to use clinical characteristics to distinguish between patients with COVID-19 having malaria-like symptoms. Detection of IgM using RDTs may be useful in predicting CT values for SARS-CoV-2 real-time PCR and therefore transmission.

## Background

Malaria is a major infectious disease and the most common diagnosis for febrile patients [1] It is endemic in Ghana and globally there is an estimated 241 million malaria cases in 2020 in 85 malaria endemic countries with most originating from countries in the WHO African regions [2]. Deaths associated with malaria increased by 12% in 2020 as compared to the previous years due to COVID-19 pandemic which disrupted management of malaria [2].

Some infectious diseases including dengue fever, zika and chikungunya in the tropics mimic malaria and the similarity in clinical presentation makes diagnosis difficult [3]. Both malaria and COVID-19 share some symptoms such as fever, fatigue and headache [4, 5]. In less developed countries including Ghana, there is limited access to advanced diagnostic tools to clearly detect and differentiate between non-plasmodium malaria-like illnesses Thus, clinicians rely mainly on clinical presentations to inform prompt management. Failure to differentiate COVID-19 at the primary health care may cause delays in management, prolong hospitalization, potential transmission of SARS-CoV-2 especially among hospitalized patients and increase the possible emergence of new variants [6]. Accurate detection of COVID-19, as well as malaria, is critical for proper health care [7], hence there is the need to investigate COVID-19 in patients presenting with malaria-like symptoms at primary health care facilities.

This study investigated the occurrence of COVID-19 in patients presenting with malaria-like symptoms at a primary healthcare in Accra, Ghana. The anti-SARS-CoV-2 presence was determined in patients, and the possible effects of SARS-CoV-2 infection on haematological parameters to aid in accurate and early diagnosis, investigated.

## Materials and methods

### Study site and population

The cross-sectional study was conducted from 8^th^ June to 2^nd^ August, 2021 at Korle Bu Polyclinic, Accra. The Korle Bu Polyclinic is a government polyclinic in Greater Accra Region in Ghana with coordinates (5.542078; -0.23448). It is a major primary health care center which is administratively linked to the Korle Bu Teaching hospital, a major tertiary healthcare facility in Accra, Ghana. Furthermore, it offers primary healthcare services to the Korle Bu community, its environs, and the city Accra as a whole. All consenting adults (≥18 years) who presented with malaria-like symptoms including malaise, general body pains, general body weakness, and headache and +/- fever (37.5°C), were enrolled into the study. A questionnaire was administered to obtain socio-demographic information and clinical history including the symptoms patients presented with. Simple random sampling was employed in selection of study participants to avoid bias until the total sample size of 300 was obtained (105 males and 195 females). All participants were recruited to the study from 8th June to 2nd August, 2021 during whichnasopharyngeal swabs, oropharyngeal swabs, whole bloodand data were collected.

### SARS-CoV-2 RNA detection and genomic sequencing

Naso- and oropharyngeal swabs and whole blood was collected from enrolled participants. The swabs were then transported to the Noguchi Memorial Institute for Medical Research (NMIMR), department of Virology for RNA extraction using SPIN-X Viral RNA extraction kit (SD BIOSENSOR, Inc, Hayana, India; Cat. No 11SPN10) and RT-PCR testing using Mico Biomed VERI-Q Prep kit (MiCo BioMed Co., Ltd; Apr. 2020, Korea; Cat. No 7K401) with sensitivity of 96.4% and specificity of 96.4% was performed according to the manufacturer’s instructions. The swab samples were also used for the COVID-19 Antigen Test using SARS-CoV-2 Antigen Test Kit (Colloidal Gold Chromatographic Immunoassay) (Ultra-Diagnostics-Bio, China; Lot. No 20102001) with sensitivity over 90% and specificity over 99% to detect SARS-CoV-2 Nucleocapsid (N) protein as described by Terpos *et al*., [8]. SARS-CoV-2 genomic sequencing was performed by the genomic surveillance teams at NMIMR using MinIon sequencing and was supported by the CSIGN project (Virology Department, NMIMR).

COVID-19 positive samples with cycle threshold values below 30 were selected and RNA extraction was done using Qiagen extraction kit (Qiagen, USA; 52904). cDNA preparation was performed using Lunascript RT Supermix with cyclical conditions of 25°C for 2 mins, 55°C 10 mins, 95°C for 5 mins. Master mix was prepared using odd and even different primer set (Pool 1 and Pool 2) with the cDNA template for PCR with cyclical conditions of 98°C for 30 seconds, 98°C for 15 mins, 65°C for 5 mins and the reaction was subjected to 35 cycles. All amplicon pools (2 μl each) was quantify using the Qubit ds DNA Broad range kit (Thermo Fisher Scientific, USA; Q32850). End preping (repairing 3’ and 5’ blunt ends), barcoding (identification) and clean up (using beads to remove primer dimers) was done. Ligation buffer, ligation enzyme and Adapter (Oxford Nanopore Technologies, Oxford Science Park, UK) were mix to eluent to form the library prep and then loaded into the flow cell. After the flow cell run was complete, the Maximum Likelihood method and Tamura-Nei model [9] was used to infer the evolutionary history of the lineages. The tree that gave the highest log likelihood of -41598.42 is shown. Initial tree(s) for the heuristic search were obtained automatically by applying Neighbor-Join and BioNJ algorithms to a matrix of pairwise distances estimated using the Tamura-Nei model, and then selecting the topology with superior log likelihood value. A discrete Gamma distribution was used to model evolutionary rate differences among sites (5 categories (+G, parameter = 0.0500)). The rate variation model allowed for some sites to be evolutionarily invariable ([+I], 49.77% sites). The tree is drawn to scale, with branch lengths measured in the number of substitutions per site. Codon positions included were 1st+2nd+3rd+Noncoding. There were a total of 29611 positions in the final dataset. The analysis was performed using MEGA11 [10]. The final annotation of the tree was performed in iTOL v6 (https://itol.embl.de/). Accession numbers in GISAID are EPI_ISL_8065649, EPI_ISL_8065647, EPI_ISL_8065673, EPI_ISL_8065670, EPI_ISL_8065654, EPI_ISL_8065663, EPI_ISL_8065652, EPI_ISL_8065651, EPI_ISL_8065662, EPI_ISL_8065672, EPI_ISL_8065648, EPI_ISL_8065650, EPI_ISL_8065653, EPI_ISL_8065671, EPI_ISL_8065655, EPI_ISL_8065674 and EPI_ISL_8065656.

### Malaria diagnosis by Rapid Diagnostic Test (RDT), microscopy and nested conventional PCR

The blood samples were used to perform three diagnostic tests which include Malaria RDT, microscopy and nested PCR. SD Bioline Malaria Antigen *Plasmodium falciparum* test kit (SD Bioline, St Louis USA; Lot. No. 05FK50) with sensitivity of 99.7% and specificity of 99.5% was used to detect *Plasmodium falciparum* as previously described by Tangpukdee *et al* [11]. For the detection and speciation of *Plasmodium falciparum*, a thick blood smear was prepared and stained using 10% Giemsa stain (1 ml of Giemsa to 9ml of distilled water) as previously described by Abuaku *et al* [12]. The total number of 200 white blood cells was counted and the corresponding parasites count was recorded and used to estimate the parasite density.

Chelex DNA extraction from blood spots on Whatman’s filter paper (Diagger Scientific Inc, USA; B07D21FRRM) was performed using the saponin and chelex reagents following manufacturer’s protocol (SIGMA, Saint Louis, MO 63103, USA; Product. No. 84510). Nested PCR was performed to detect *Plasmodium* species in the DNA extract using two primer sets (Table 1). In Nest I, 3μl of DNA template was added to 7.5μl DreamTaq master mix following manufacturer’s protocol with 0.4μl of each rPLU5 and rPLU6 to form 15 μl reaction to detect 18S rRNA genes. Polymerase chain reaction was performed using a thermocycler (Applied Biosystems™ SimpliAmp™) with cyclical conditions as previously described by Li *et al*. [11] but was optimized to (94°C for 2 min, 94°C for 30 seconds, 54°C for 1 min, 68°C for 1 min, 5 min final extension at 68°C and the reaction was subjected to 35 cycles). Amplicons as a result of Nest I was used as a template for Nest II. In Nest II, 2μl of Nest I PCR product was added to 7.5μl of the DreamTaq master mix, 6.20μl of double distilled water, 0.4μl each of rFAL2 and rFAL3 to form a 16.5 μl reaction to detect merozoite surface protein 2 (msp2) which is a specific target sequence for *Plasmodium falciparum* and has a band size of 200 base pairs. The cycling conditions for Nest II was the same as that used for Nest I with a slight modification in the annealing temperature of 59°C instead of 54°C for 1 min. Size-fraction of the PCR products was performed on 2% agarose gel stained with Ethidium bromide (Biotium, Hayward, California, USA). The gel was run at 130V for 45 mins and visualized under ultraviolet light.

**Table 1:** Primers sequences for *Plasmodium* parasite identification.

Primer	Sequence: 5’-3’
rPLU5	CCTGTTGTTGCCTTAAACTTC
rPLU6	TTAAAATTGTTGCAGTTAAAACG
rFAL2	ACACAATGAACTCAATCATGACTACCCGTC
rFAL3	TTAAACTGGTTTGGGAAAACCAAATATATT

### Detection of anti-SARS-CoV-2 antibody

Genrui 2019-nCoV IgG/IgM detection kit (Colloidal Gold-Based) (Lot. No 52027072) with sensitivity of 91.01% and specificity of 95.37% was used to detect specific IgM/ IgG antibodies against COVID S protein following manufacturer’s instructions.

### Full blood count analysis

Two to three millilitres of venous blood was collected into Ethylenediamine tetraacetic acid (EDTA) tube and then analyzed using the Mindray Hematology analyser (Medsinglong CO. LTD, Guangdong, China).

### Data management and statistical analysis

Data was collected into data sheets and notebook on the field and laboratory. Data were later entered, stored and managed in Microsoft Excel, 2019. The corresponding author and the first author only had access to information that could identify individual participants during or after data collection. A test for normality using the Shapiro-Wilk test showed non-normal distributions and so non-parametric analyses were done. Proportions and percentages analysis was done using Excel. Paired T-test was also used to determine whether there was a significant difference between symptoms presented by COVID-19 patients before entry and at the primary health care. Chi-square test was used to determine whether there is a statistical significant relationship between the categorical variables. Pearson’s correlation test was used to determine the association between two continuous variables via SPSS version 2 (SPSS Inc., Chicago, IL). The effects of COVID-19 on haematological parameters were determined using the Chi-square test.

### Ethics

Ethical approval was sought from the Ethics and Protocol Review Committee (EPRC) of the College of Health Sciences (CHS) of the University of Ghana with protocol identification number: CHS-Et/M.3 -4.5/2020-2021 and the Ghana Health Service Ethics Review Committee with protocol identification number: GHS-ERC 028/03/21. Administrative approval letter from Korle Bu Teaching Hospital (KBTH-ADM/00652/2021) to conduct the study was also obtained. Participant Information sheet and Informed written consent forms were provided for all participants recruited into the study. All consent forms were written, hard copy and explained verbally to each participant in either English or local languages (Twi or Ga) and upon agreement each participant signed before data and clinical samples were collected. All consent forms have been well organised and securely stored and kept in the laboratory.

## Results

### Demographic and clinical characteristics of malaria patients

Among the 300 participants recruited, the prevalence of malaria by microscopy, RDT and nested PCR were 7/300 (2.3%), 7/300 (2.3%) and 8/300 (2.7%) respectively. Majority of the malaria patients were females and most came from the urban areas. Two coinfections of malaria and COVID-19 were reported (2/300; 0.7%). The average temperature recorded was 37.3°C. Majority of the participants with malaria were between the age range of 21–40 years (6/8; 75%) and the highest education level were senior high school (SHS) (4/8; 50%). The most common symptoms observed among all participants were headache, body weakness and body pain (8/8; 100%). Hypertension was the only co-morbidity reported (1/8; 12.5%) (Table 2).

**Table 2:** Demographics and medical history of patients with malaria.

Infections	Frequency (%)
Gender	Females (6/8; 75%) and males (2/8; 25%)
Age	Highest age was 53 years,
	Lowest was 18 years,
	Majority were within the age range of 21–40 years (6/8; 75%).
Marital Status	Majority were single (7/8; 87.5%).
Occupation	Majority work in the public sector (3/8; 37.5%),
	Students (2/8; 25%),
	Traders / business men and women (2/8; 25%) and
	Artisan (1/8; 12.5%).
Highest Educational level	Most were SHS (4/8; 50%),
	Tertiary students (2/8; 25%),
	Primary and (1/8; 12.5%).
	Middle school Form 4 (1/8; 12.5%).
Districts	Most came from the Ablekuma South district (5/8; 62.5%).
Temperature	Highest temperature reported was 38.8°C and lowest is 35.6°C
Co-morbidities	Hypertension was reported (1/8; 12.5%)

### Demographic and clinical characteristics of COVID-19 patients

Out of 300 study participants, 26 (8.7%) were positive for SARS-CoV-2 using the SARS-CoV-2 Antigen Test while, 60 (20%) tested positive using RT-PCR test. Majority of the COVID-19 patients were females (42/60; 70%) between the ages of 21–40 years (42/60; 70%) and the mean age was 44 years. Some participants had no educational background (20/60; 33.3%) and were traders/ businessmen and women (20/60; 33.3%). Most of the confirmed COVID-19 patients came from urban areas (Table 3).

**Table 3:** Demographics of COVID-19 patients.

Characteristics	COVID-19 Status			
	Positive (%)	Negative (%)	X^2^ value	P value
**Age groups**			0.357	0.94
18–20 years	4/60 (6.7)	16/240 (6.7)		
21–40 years	22/60 (36.7)	99/240 (41.3)		
41–60 years	19/60 (31.7)	73/240 (30.4)		
Above 60 years	15/60 (25)	52/240 (21.7)		
**Sex**			0.899	0.34
Female	42/60 (70)	154/240 (64.2)		
Male	18/60 (30)	86/240 (35.8)		
**Marital Status**			3.3	0.76
Married	31/60 (51.7)	110/240 (45.8)		
Single	22/60 (36.7)	100/240 (41.7)		
Widowed	7/60 (11.7)	23/240 (9.6)		
Separated		6/240 (2.5)		
Divorced		1/240 (0.4)		
**Highest Educational Level**			13.13	0.15
No educational background	20/60 (33.3)	65/240 (27.1)		
Tertiary level	18/60 (30)	69/240 (28.8)		
Senior High School level (SHS)	11/60 (18.3)	47/240 (19.6)		
Junior High School level (JHS)	10/60 (16.7)	23/240 (9.6)		
Primary level	1/60 (1.7)	9/240 (3.8)		
Form 4		26/240 (10.8)		
Form 5		1/240 (0.4)		
**Occupation**			25.01	0.33
Public sector	11/60 (18.3)	53/240 (22.08)		
Students	9/60 (15)	20/240 (8.33)		
Unemployed	14/60 (23.3)	35/240 (14.6)		
Artisans	6/60 (10)	33/240 (13.8)		
Traders/ Business men and women	20/60 (33.3)	97/240 (40.4)		
Retired	None	97/240 (40.4)		
**Residence**	Urban areas in Accra	Urban areas in Accra		

There were 35 out of 60 COVID-19 patients who had normal temperature ranging from 35.6°C -37.5°C representing 58%. The mean temperature was 37.2°C. The highest comorbidity reported was hypertension (9/60; 15%) (Table 4). The most common symptom experienced by the COVID-19 patients was headache (95%; 57/60) (Table 4). Negative correlation was reported between the cumulative number of symptoms and Cycle threshold (CT) values of both the ORF3a and N protein of COVID-19 patients, *r* (58) = -0.744, p < 0.001 and *r* (58) = -0.754, p <0.001 respectively (Table 5). This revealed that majority of COVID-19 patients with CT values below 30 (higher level of viral RNA) (67.7%; 21/31) presented with 5 or more symptoms.

**Table 4:** Medical history of COVID-19 patients.

Clinical Characteristics*	COVID-19 Status			
	Positive (%) N = 60	Negative (%) N = 240	X^2^ value	*P* value
**Average Temperature**	37.2 °C	36.9°C	18.10	0.92
**Co-morbidities**			13.62	0.12
Hypertension	9/60 (15)	42/240 (17.5)		
Diabetes	2/60 (3.3)	15/240 (6.3)		
Asthma	1/60 (1.7)	8/240 (3.3)		
Hypertension and diabetes	4/60 (6.7)	12/240 (5)		
Sickle cell diseases	None	1/240 (0.41)		
**Clinical Symptoms**				
Headache	57/60 (95)	211/240 (87.9)	2.85	0.240
General body weakness and pain	47/60 (78.3)	173/240 (72.1)	5.78	0.005
Tiredness	36/60 (60)	158/240 (65.8)	14.27	0.001
Fever	25/60 (42)	100/240 (41.6)	8.811	0.012
Cough	18/60 (30)	61/240 (25.4%)	15.64	0.001
Bitter taste	22/60 (36.7)	42/240 (17.5)	22.16	0.001
Sore throat	11/60 (18.3)	37/240 (15)	15.83	0.001
Loss of taste	5/60 (8.3)	15/240 (6.2)	15.56	0.001
Loss of smell	6/60 (10.0%)	4/240 (1.7)	21.65	0.001
Diarrhoea	2/60 (3.3%)	5/240 (2.1)	16.51	0.001
Difficulty in breathing	1/60 (1.7%)	13/240 (5.4)	19.97	0.001
Others (included cold, dizziness and chills.	7/60 (11.7%)	26/240 (10)	26.00	0.130

*Inclusive of malaria infections

**Table 5:** Correlation of the cumulative number of symptoms of COVID-19 patients and cycle threshold value the Open Reading Frame 3a (ORF3a) and N gene.

		Cumulative number of symptoms	ORF3a	N
**Cumulative number of symptoms**	Pearson Correlation	1	-.744**	-.754**
	*P value* Sig. (2-tailed)		.000	0.00
	N	60	60	60

**. Correlation is significant at the 0.01 level (2-tailed)

The white blood cell, neutrophil, lymphocyte, monocyte, eosinophil, basophil, red blood cell, and platelet counts were similar for patients with and without COVID-19 (p.0.05), excluding all malaria infections from the analysis.

### SARS-CoV-2 lineage, antibody presence and vaccination status

**Fig 1:**
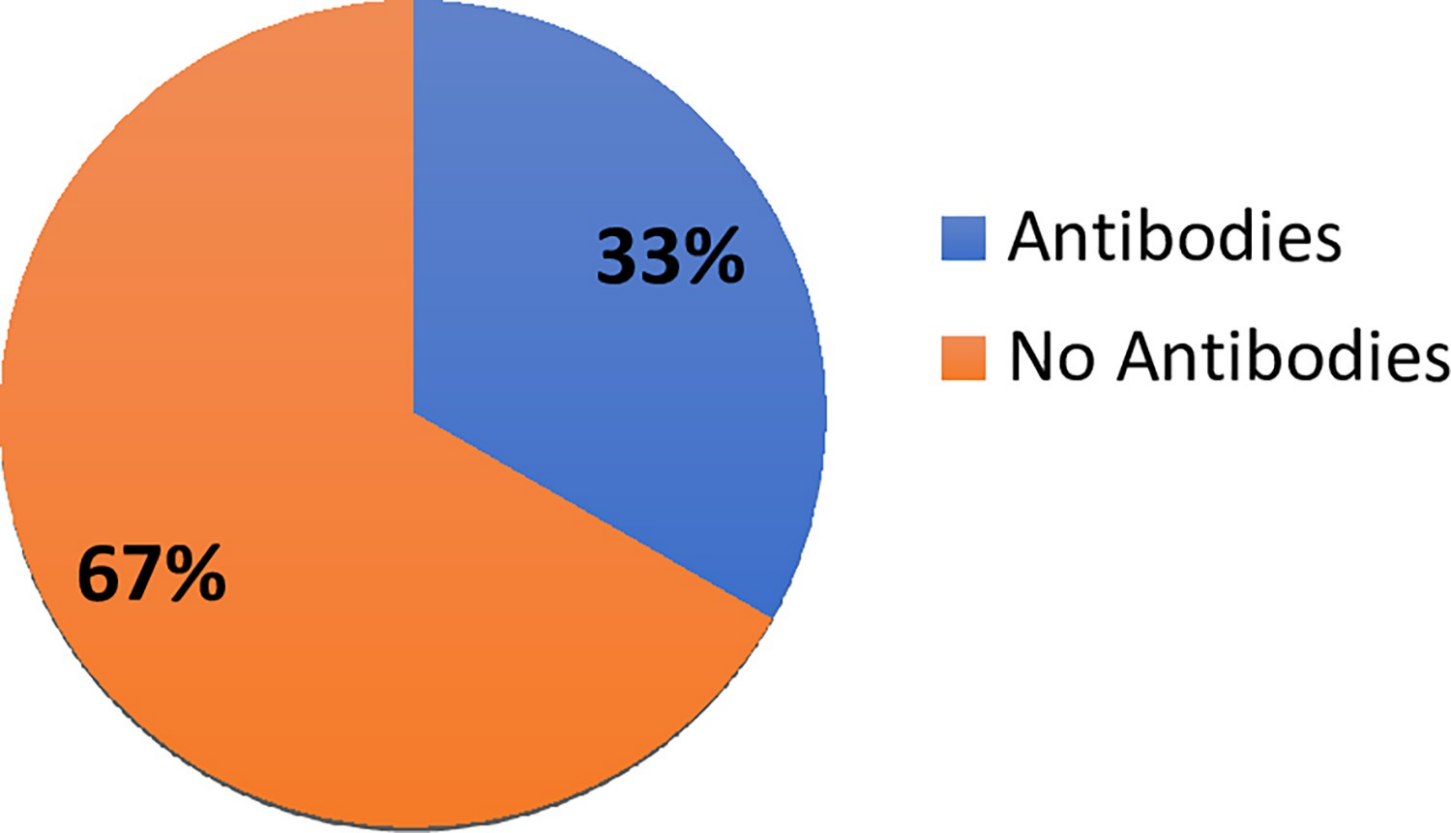
SARS-CoV-2 antibody status of AstraZeneca vaccinated persons.

**Fig 2:**
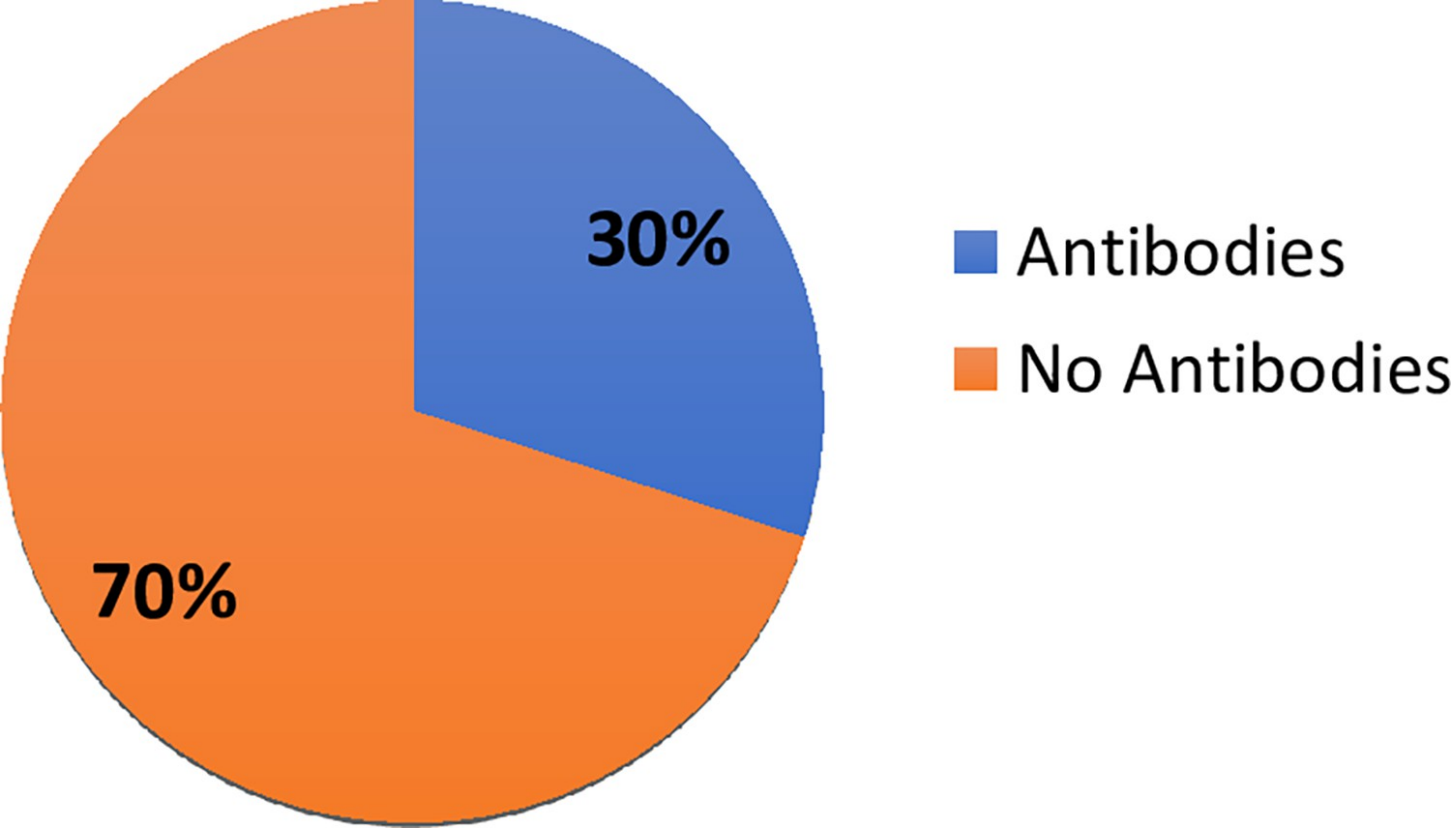
SARS-Cov-2 IgG antibody status of vaccinated persons with single dose.

**Fig 3:**
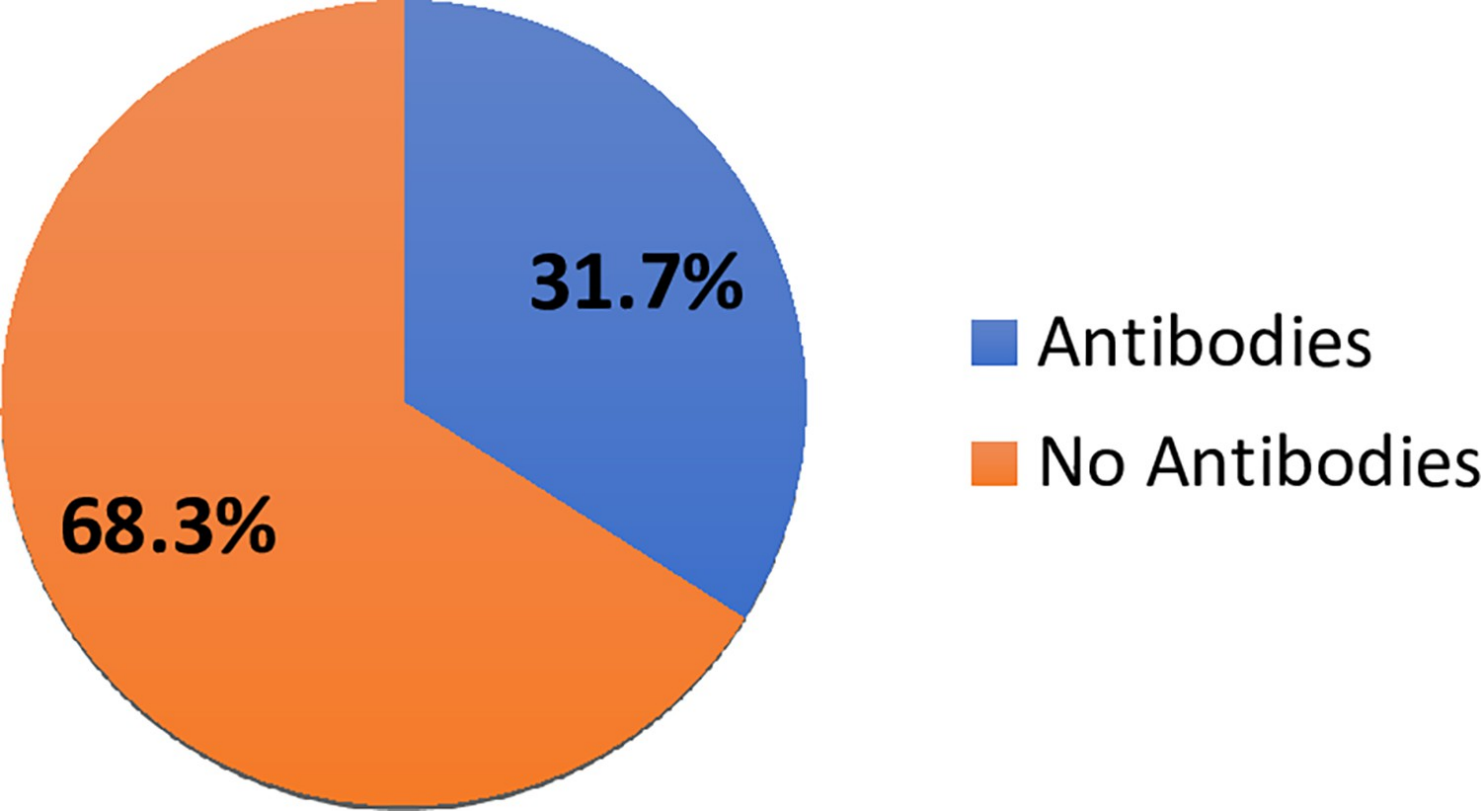
SARS-Cov-2 IgG antibody status of vaccinated persons with double dose.

Measurement of SARS-CoV-2 antibodies in the study participants showed that 32% (113/300) of the study patients had COVID-19 IgG antibody, 12% (36/300) had COVID-19 IgM and 50.3% (151/300) had no COVID-19 IgM and IgG. There were 28 out of 60 (46.7%) COVID-19 patients who had COVID-19 IgG antibody, 45% (27/60) had COVID-19 IgM and 30% (18/60) had no antibodies. There were 51 Astrazeneca vaccinated patients (single and double dose) out of 300 study patients and 33.3% (17/51) had IgG antibodies (Fig 1). There were 10 out of 51 vaccinated patients who had a single dose of the Astrazeneca vaccine and 30% (3/10) showed IgG antibodies (Fig 2). Moreover, 80% (41/51) out of 51 vaccinated patients had the double dose of vaccination and 31.7% (13/41) showed IgG antibodies (Fig 3). Eighteen out of 51 showed evidence of their vaccination cards which included the dates and details of vaccine.

There was significant association between COVID-19 IgM and ORF3a (*X*^*2*^ = 17.475; *df* = 1; *P* < 0.001) and N gene of PCR (*X*^*2*^ = 19.862; *df* = 1; p < 0.001). This revealed that majority of confirmed COVID-19 IgM had low CT values (CT < 30) (81% and 92.6%) (Table 6).

**Table 6:** COVID-19 IgM and PCR CT values of ORF3a and N gene.

IgM	ORF3a gene		N gene	
	Low CT	High CT	Low CT	High CT
**COVID-19 IgM Positive**	81% (22/27)	18.5% (5/27)	92.6% (25/27)	7.4% (2/27)

**Fig 4:**
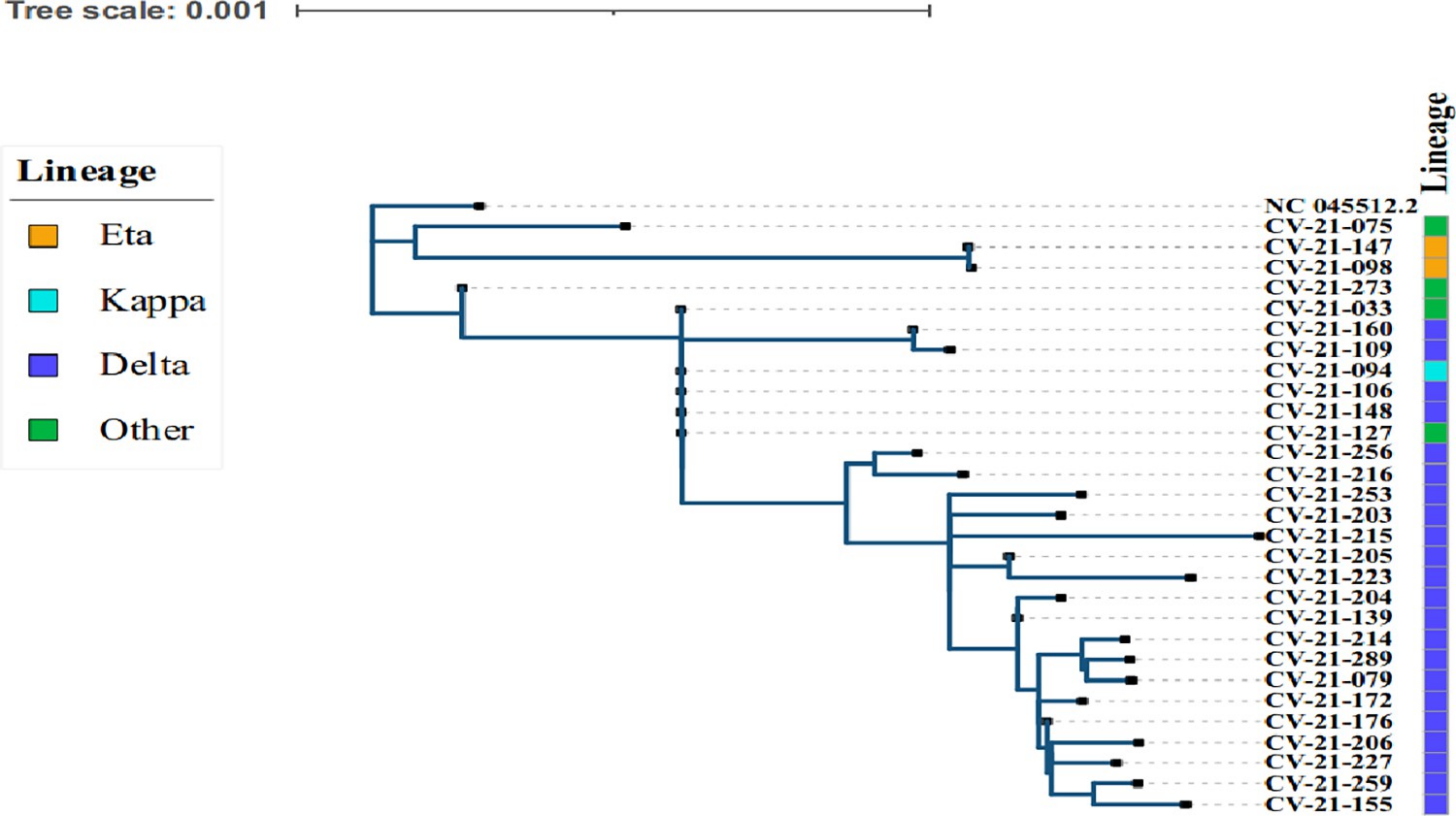
Evolutionary linages (variants) of SARS-CoV-2 in COVID-19 patients.

SARS-CoV-2 positives with PCR CT values below 30 were selected for genomic sequencing and 23 out of 25 selected CT values reported Delta variants with majority originating from B.1.617.2 and AY39 lineages (Fig 4). Eta variants were reported in 2 out of 25 selected CT values (Fig 4). We used the maximum likelihood method and Tamura-Nei model (12) to infer the evolutionary history of the lineages. A discrete Gamma distribution was used to model evolutionary rate differences among sites. The analysis was performed using MEGA11 [10]. The final annotation of the tree was performed in iTOL v6 (https://itol.embl.de/) (Fig 4).

## Discussion

Malaria contributes significantly to morbidity and mortality in public healthcare facilities in Ghana. Due to the onset of COVID-19 pandemic, provision of clinical services for malaria in most moderate and high malaria burden countries were interrupted [2], and this increased the number of malaria cases. Studying factors that could contribute to the dynamics of the pandemic is very essential. In this study, COVID-19 was investigated in patients who presented with malaria-like symptoms at the KPFMD, a primary care health facility Korle Bu polyclinic in Accra. Some of the patients who presented with malaria-like symptoms had COVID-19 using PCR and may have been treated for only malaria based on their symptoms even though they harboured SARS-CoV-2. Some of the study patients with malaria-like symptoms were also reactive for SARS-CoV-2 IgG antibody. Headache, general body weakness, general body pains and tiredness were the top four commonest symptoms reported. Majority of the COVID-19 patients had normal lymphocytes and platelets count. Since there were no clear-cut symptoms suggesting COVID-19, the issue of differential diagnosis becomes difficult when considering individuals reporting with malaria-like symptoms. This is even made more difficult because of the relatively few malaria cases detected in this study.

These findings are similar to a study conducted in India which reported 10.34% of COVID-19 among febrile patients at a malaria clinic [7]. Most febrile patients in India disagreed for nasopharyngeal and oropharyngeal swabs to be taken and this could contribute to the low prevalence of COVID-19 reported. Also, the anxiety and fear of COVID-19 was alarming during the peak of the pandemic and most people were unwilling to attend to healthcare facilities and this led to fewer clinic visits.

Monitoring patient’s temperature is important however, measuring temperature alone is not informative enough because >50% of participants in our study who had normal temperature (< 37.5°C) or were afebrile did not exclude the possibility of COVID-19. A similar finding was also reported in a study by Mo and others, where about 8.7% of COVID-19 patients had no fever at admission [13].

Some of the study participants with COVID-19 (46%) were reactive for SARS-CoV-2 IgG antibody which indicates previous exposure to SARS-CoV-2. Whereas 45% of the study participants with COVID 19 were reactive for SARS-CoV-2 IgM antibody which indicates current infection to SARS-CoV-2. A study in Pakistan by Batool et al., (2021), reported a similar finding of seroprevalence of SARS-CoV-2 IgG antibody (33%) among healthcare workers [14]. Our study showed that, majority of COVID-19 patients (92.6%) who were reactive for SARS-CoV-2 IgM antibody had PCR CT< 30 and this key finding confirmed that antibody test can also be used as diagnostic and surveillance tool to assist screening of COVID-19 to provide vital information on the spread of the disease and to forecast future hotspots areas.

Not all Astrazeneca vaccinated patients had attained the full immune protection against SARS-CoV-2 hence they are at risk of being infected. The need to understand antibody responses in such patients and to continuously observe all the COVID-19 protocols even after vaccination, is emphasized

Hematological parameters and COVID-19 disease are clinical markers related to disease severity and progression. Most COVID-19 participants, malaria participants and participants with no infections had normal full blood counts counts and this suggests that most of the confirmed COVID-19 patients had mild to moderate infection. These findings are in line with another study by Araya et al. [15] and Y. Shang et al. [16] where majority (80.9%) of the COVID-19 patients presented with mild disease. Araya Wordofa [15] and Liu Zhang [17] reported that severe COVID-19 patients are more likely to have low lymphocyte and platelet counts than those with mild and moderate COVID-19 infection and this suggests that majority of confirmed COVID-19 patients in the current study had mild to moderate infections.

## Conclusion

In summary, it is evident from the present study that both COVID-19 and malaria share some common clinical symptoms during the clinically symptomatic stage of infection which could lead to misdiagnosis. Due to the similarity of symptoms, screening for COVID-19 in patients presenting with malaria-like symptoms is vital for immediate diagnosis and treatment to avoid serious complications due to the non-specific and wide range of signs and symptoms during the clinically symptomatic stage of infection.

## References

[1] Stoler J, Awandare GA. Febrile illness diagnostics and the malaria-industrial complex: a socio-environmental perspective. BMC Infect Dis. 2016;16(1):683. doi: 10.1186/s12879-016-2025-x 27855644 PMC5114833

[2] World Health Organisation (2021). World Malaria Report. Geneva. 1–322

[3] Yong LS, Koh KC. A case of mixed infections in a patient presenting with acute febrile illness in the tropics. Case Rep Infect Dis. 2013;2013:562175. doi: 10.1155/2013/562175 23533853 PMC3600219

[4] Chanda-Kapata P, Kapata N, Zumla A. COVID-19 and malaria: A symptom screening challenge for malaria endemic countries. Int J Infect Dis. 2020;94:151–3. doi: 10.1016/j.ijid.2020.04.007 32344326 PMC7184246

[5] Sherrard-Smith E, Hogan AB, Hamlet A, Watson OJ, Whittaker C, Winskill P, et al. The potential public health consequences of COVID-19 on malaria in Africa. Nat Med. 2020;26(9):1411–6. doi: 10.1038/s41591-020-1025-y 32770167 PMC7613562

[6] Nunthavichitra S, Prapaso S, Luvira V, Muangnoicharoen S, Leaungwutiwong P, Piyaphanee W. Case Report: COVID-19 Presenting as Acute Undifferentiated Febrile Illness-A Tropical World Threat. Am J Trop Med Hyg. 2020;103(1):83–5. doi: 10.4269/ajtmh.20-0440 32419694 PMC7356458

[7] Guha SK, Biswas M, Gupta B, Acharya A, Halder S, Saha B, et al. A report on incidence of COVID-19 among febrile patients attending a malaria clinic. Trop Parasitol. 2021;11(1):38–41. doi: 10.4103/tp.TP_105_20 34195059 PMC8213116

[8] Terpos E., Ntanasis-Stathopoulas L and Skvarc M. Clinical application of a New SARS-CoV-2 Antigen Detection Kit (colloidal Gold) in the Detection of COVID-19. 2021;11(6): 995. doi: 10.3390/diagnostics11060995 34070844 PMC8229208

[9] Tamura K. and Nei M. Estimation of the number of nucleotide substitutions in the control region of mitochondrial DNA in humans and chimpanzees. Molecular Biology and Evolution.1993; 10:512–526 doi: 10.1093/oxfordjournals.molbev.a040023 8336541

[10] Tamura K., Stecher G., and Kumar S. MEGA 11: Molecular Evolutionary Genetics Analysis.2021; Version 11. Molecular Biology and Evolution 10.1093/molbev/msab120 PMC7086165 31904846

[11] Tangpukdee N, Duangdee C, Wilairatana P, Krudsood S. Malaria diagnosis: a brief review. Korean J Parasitol. 2009;47(2):93–102. doi: 10.3347/kjp.2009.47.2.93 19488414 PMC2688806

[12] Abuaku B., Amoah L.E., Peprah Y., Asamoah A., Amoako E.O., Donu D., et al. Malaria parasitaemia and Mrdt diagnostic performances among symptomatic individuals in selected health care faciltities across Ghana. BMC Public Health. 2021; 21:239 33509161 10.1186/s12889-021-10290-1 PMC7844948

[13] Mo P, Xing Y, Xiao Y, Deng L, Zhao Q, Wang H, et al. Clinical Characteristics of Refractory Coronavirus Disease 2019 in Wuhan, China. Clin Infect Dis. 2021;73(11):e4208–e13 doi: 10.1093/cid/ciaa270 32173725 PMC7184444

[14] Batool H, Chughtai O, Khan MD, Chughtai AS, Ashraf S, Khan MJ. Seroprevalence of COVID-19 IgG antibodies among healthcare workers of Pakistan: a cross-sectional study assessing exposure to COVID-19 and identification of high-risk subgroups. BMJ Open. 2021;11(8):e046276. doi: 10.1136/bmjopen-2020-046276 34400447 PMC8370836

[15] Araya S, Wordofa M, Mamo MA, Tsegay YG, Hordofa A, Negesso AE, et al. The Magnitude of Hematological Abnormalities Among COVID-19 Patients in Addis Ababa, Ethiopia. J Multidiscip Healthc. 2021;14:545–54. doi: 10.2147/JMDH.S295432 33688198 PMC7936683

[16] Shang Y., Pan C., Yang X., Zhong M., Shang X., Wu Z., et al. Management of critically ill patients with COVID-19 in ICU: statement from front-line intensive care experts in Wuhan, China. *Ann Intensive Care*, 2020 10(1), 73. doi: 10.1186/s13613-020-00689-1 32506258 PMC7275657

[17] Liu X, Zhang R, He G. Hematological findings in coronavirus disease 2019: indications of progression of disease. Ann Hematol. 2020;99(7):1421–8. doi: 10.1007/s00277-020-04103-5 32495027 PMC7266734

